# The Effect of COVID-19 Vaccines on Hospital Admission and Severity of Symptoms Among COVID-19 Patients in Saudi Arabia, 2021

**DOI:** 10.7759/cureus.41067

**Published:** 2023-06-28

**Authors:** Reem F Alnemari, Fawziah A Roublah, Amina A Bargawi

**Affiliations:** 1 Preventive Medicine, Ministry of National Guard Health Affairs, King Abdullah International Medical Research Center, King Saud bin Abdul-Aziz University for Health Sciences, Jeddah, SAU

**Keywords:** symptom severity, hospital admission, astrazeneca, pfizer, vaccine, covid-19

## Abstract

Introduction

Following the World Health Organization (WHO) declaration of coronavirus disease 2019 (COVID-19) as a pandemic, Saudi Arabia took unpreceded precautions to prevent and control the spread of the severe acute respiratory syndrome coronavirus 2 (SARS‑CoV‑2) infection. It is one of the first countries in the world to grant the authorization to use the Pfizer-BioNTech vaccine. This study aimed to assess the effect of one dose of COVID-19 vaccines (Pfizer-BioNTech, Manhattan, New York City*,* and Oxford-AstraZeneca, Cambridge, United Kingdom) among the Saudi population regarding symptom severity, hospital admission rate, and death.

Methods

An observational retrospective cohort study was conducted using data from COVID-19 surveillance records at King Abdulaziz Medical City (KAMC), Saudi Arabia, from January to May 2021. All confirmed COVID-19 patients who had positive tests by reverse transcription polymerase chain reaction (RT-PCR) assay of a nasopharyngeal swab were included in the study. Patients diagnosed outside KAMC and cases below 18 years old were excluded from the study. The research was approved by King Abdullah International Medical Research Center (NRJ21J/303/12). Multivariable logistic regression was conducted to estimate the odds of hospitalization among vaccinated and unvaccinated patients.

Results

A total of 1058 cases were included in the analysis. Two hundred sixty-five (265; 25%) patients were vaccinated with one dose of either Pfizer-BioNTech or Oxford Astra-Zeneca, and 793 (75%) were unvaccinated. The median age was 34 (IQR 25-51), primarily Saudi (94.6%) and male (59.5%). The odds of being vaccinated (CI: 1.284-2.882, P 0.002) were 1.924 times greater for males than females. Young patients below 40 had 1.997 times higher odds (CI: 1.238-3.222, P 0.004) of being vaccinated than patients above 60. The hospital admission rate was low among both groups (12.9%); however, it was significantly lower among the vaccinated group (2.3%) as compared to the unvaccinated (16.5%). The results showed significant differences in symptom severity among the groups. For vaccinated, only one patient (0.4%) died, one patient was admitted to the ICU, and one patient (0.4%) was admitted to the hospital isolation ward. On the contrary, among the unvaccinated group, 19 patients (2.4%) died, 17 patients (2.1%) were admitted to the ICU, and 114 patients (14.4%) were admitted to the hospital isolation ward.

Conclusion

This study demonstrates that one dose of COVID-19 vaccines, either Pfizer-BioNTech or Oxford-AstraZeneca, reduced the probability of death by 2% and hospital admission by 15% before the spread of the Delta variant (B.1.617). For generalizable results, nationwide studies using national surveillance data are recommended to assess multiple doses efficacy on different variants of the SARS-CoV-2 infection.

## Introduction

The first confirmed case of severe acute respiratory syndrome coronavirus 2 (SARS‑CoV‑2) reported in Saudi Arabia was on March 2, 2020 [1]. The government was proactive in preventing coronavirus disease 2019 (COVID-19) after the first infected cases appeared. Several precautions were implemented such as travel restrictions on international and internal flights, lockdowns of mosques, schools, universities, and shopping malls, and suspension of attendance of employees in the government and private sectors, followed by a complete curfew. Furthermore, the Umrah suspension was imposed on March 4, 2020, the Ministry of Hajj called on countries to postpone bookings for the 2020 Hajj season in early April, and the Hajj was restricted to local COVID-19 recovered cases [2,3].

SARS-CoV-2 has a low fatality rate compared to the two previous coronavirus epidemics caused by SARS-CoV and MERS-CoV [4]. Since its emergence, the enormous number of infected persons with severe viral pneumonia demonstrated that SARS-CoV-2 is highly contagious. Therefore, it has drawn the attention of multiple authorities in order to protect their communities and stop or slow down the transmission of this disease [4]. Evidence suggests that vaccination is the most effective method to control the spread and prevent infectious diseases [5]. Many vaccines against SARS-CoV-2 have been developed. The Pfizer/BioNTech vaccine (Manhattan, New York City) was given authorization by the U.S. Food and Drug Administration (FDA) in December 2020. The vaccine is recommended for people aged 16 and over to prevent SARS-CoV-2 infection and provides immunogenicity for at least 119 days following the first dose of immunization [6,7]. Bernal et al. assessed the effectiveness of the Pfizer-BioNTech (BNT162b2) and Oxford-AstraZeneca (ChAdOx1-S; Cambridge, United Kingdom) vaccines in the actual world in terms of confirmed COVID-19 symptoms, hospitalizations, and deaths [8]. The results showed that vaccination with either one dose of BNT162b2 or ChAdOx1-S was consistent with a considerable reduction in symptomatic COVID-19 and further protection against severe illness.

The Saudi Food and Drug Authority (SFDA) stated on December 10, 2020, that it had accepted the registration of the Pfizer-BioNTech COVID-19 vaccine in Saudi Arabia after Pfizer's request to register the vaccine so that Saudi health authorities could import and use [9]. Although Saudi Arabia was one of the first countries to conduct a COVID-19 vaccination program, limited studies estimated the efficacy of COVID-19 vaccines in Saudi Arabia. This study aimed to assess the effect of administering a single dose of Pfizer-BioNTech or Oxford-AstraZeneca among the Saudi population by comparing hospital admission rates of COVID-19 cases and symptoms severity between vaccinated and unvaccinated patients.

## Materials and methods

Study design

An observational retrospective cohort study was conducted using the data obtained from COVID-19 Clinical Assessment Team records, the surveillance team that worked during the pandemic.

Study setting 

The study was performed at King Abdulaziz Medical City (KAMC), Ministry of National Guard-Health Affairs, Western region, Saudi Arabia, from the period of January to May 2021, when the Delta variant, known as B.1.617, was realized worldwide for the first time [8,10].

Participants

The study included all confirmed COVID-19 patients who had positive tests by real-time reverse transcription-polymerase chain reaction (RT-PCR) assay of a nasopharyngeal swab. Suspected cases without confirmed results, those who were diagnosed outside KAMC, and patients below the age of 18 were excluded from the study, as vaccination wasn't introduced for children during the study period in Saudi Arabia.

Sample size

**Figure 1:**
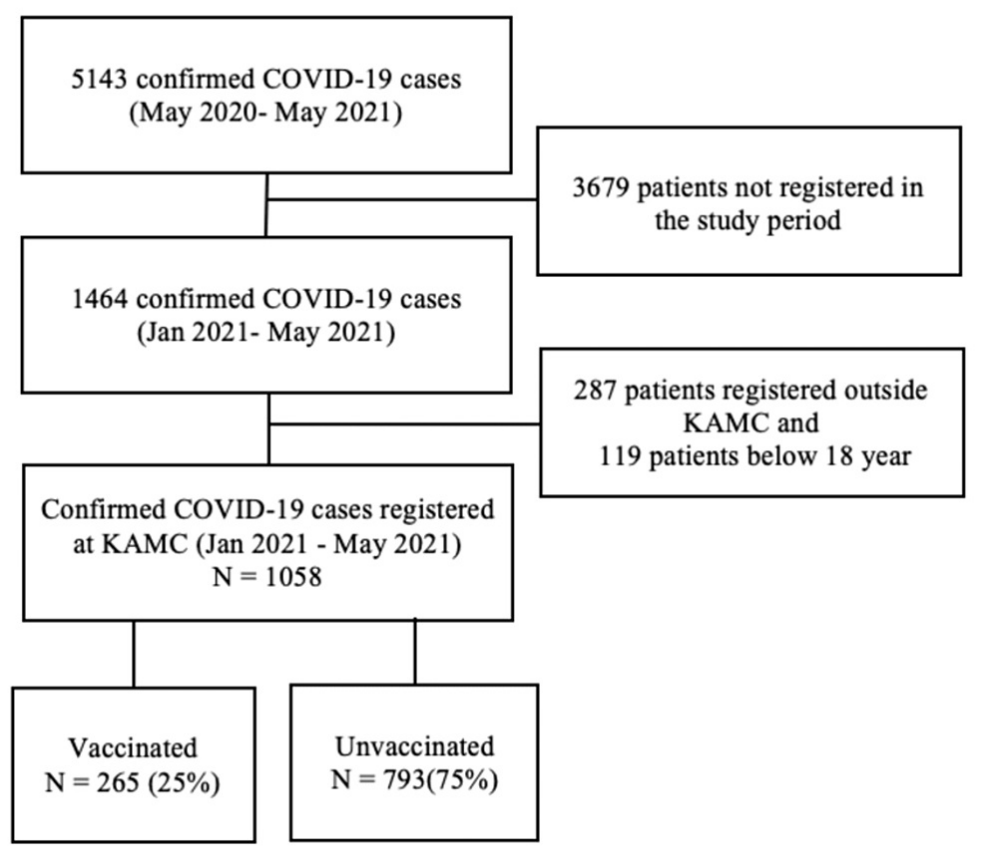
A demonstration of the number of participants included in the study

This was a consecutive sample using all recorded data. The records of the KAMC surveillance team included 5143 patients with COVID-19 from May 2020 to May 2021. Only the patients registered from January 2021 (1464 patients), when the vaccines were administered in Saudi Arabia, till May 2021 fitted for the inclusion. Out of 1464 patients, 287 were excluded because they were diagnosed outside KAMC, and 119 patients were below 18 years of age. After applying the exclusion, 1058 patients were included in the analysis (Figure 1).

Data collection 

The data were obtained from the electronic medical records and surveillance system at King Abdulaziz Medical City. Since the beginning of the pandemic, a trained team of physicians and nurses who worked as the COVID-19 Clinical Assessment Team was responsible for following all the confirmed cases diagnosed at King Abdulaziz Medical City once they received the swab results till their recovery or death. They classified the patients according to the severity of their symptoms into asymptomatic cases who were free of symptoms; mild cases who complained of mild fever, cough, or fatigue but did not have shortness of breath or abnormal chest imaging; moderate cases who had shortness of breath or their imaging showed evidence of lower respiratory disease while their oxygen saturation (SpO2) was ≥94%; severe patients who had pneumonia that required admission, and very severe cases who required ICU admission (respiratory failure, septic shock, or multiple organ dysfunction) [11]. Accordingly, the COVID-19 Clinical Assessment Team followed the cases in home isolation through regular phone calls for asymptomatic, mild, or moderate cases or arranged hospital admission at the isolation ward for severe cases or ICU for critically ill patients.

Statistical analysis

Descriptive analysis in the form of frequency and percentages was used for categorical variables while median and interquartile range (IQR) for age, as it has outliers. Chi-square statistics (χ2) were used to test the association between vaccination status and various variables, such as sociodemographic characteristics and severity of symptoms. A P-value below 0.05 was considered a statistically significant limit. Binary logistic regression was used to predict vaccination status among sociodemographic characteristics. Multivariable logistic regression was conducted to estimate the odds of hospitalization among vaccinated and unvaccinated patients in adjusting for age, gender, and morbidities. The magnitude of the predictor factors was described as an odds ratio with a 95% confidence interval. All analyses were implemented in the Statistical Package for Social Sciences (SPSS) version 25 (IBM Corp., Redmond, WA).

Ethical considerations 

The data, including the sociodemographic characteristics, past medical history, symptoms, admission status, and vaccination history, were stored at the principal investigator's office and only accessed by the authors; no names or ID numbers were recorded on the data collection form. The research was approved by the ethical and scientific committee of King Abdullah International Medical Research Center at King Abdulaziz Medical City (NRJ21J/303/12).

## Results

The analysis included 1058 patients with positive COVID-19 tests (positive nasopharyngeal swabs) from January to May 2021. Only 265 (25%) patients received one dose of mRNA Pfizer-BioNtech (BNT162b2) or AstraZeneca-Oxford (AZD1222/ChAdOx1-S) during the study duration. The demographic characteristics of the investigated patients (Table 1) show that the majority (94.6%) were Saudi, the median age was 34 years (IQR 25-51 years), 52.8% were in the young age group (18-39 years), and 59.5% were males. The sociodemographic characteristics of vaccinated and unvaccinated groups significantly differed (p-value <0.001) (Table 1). In terms of proportions, vaccinated patients were more likely to be non-Saudi (45.6%), in the middle age group (18-39) (30.9%), at a median of 36 years old, and male (30.0%).

**Table 1:** Sociodemographic characteristics Data are presented as numbers (n) and percentages (%) * Statistically significant at p < 0.05

Variables		Total n = 1058	Not Vaccinated n = 793 (75%)	Vaccinated n = 265 (25%)	Chi-square	p-value
		n (%)	n (%)	n (%)		
Nationality	Saudi	1001 (94.6%)	762 (76.1%)	239 (23.9%)	13.574	<0.001*
	Non-Saudi	57 (5.4%)	31 (54.4%)	26 (45.6%)		
Age in years	Median (IQR)	34 (51-25)	34 (50-25)	36 (46-29)	Mann-Whitney U test	0.032*
	18-39	553 (52.8%)	382 (69%)	171 (30.9%)		
	40-59	323 (30.5%)	232 (71.8%)	91 (28.2%)		
	60+	182 (17.2%)	150 (82.4%)	32 (17.6%)		
Gender	Male	630 (59.5%)	441 (70.0%)	189 (30.0%)	20.348	<0.001*
	Female	428 (40.5%)	352 (82.2%)	76 (17.8%)		

The binary logistic regression analysis in Table 2 shows that non-Saudi patients had 4.583 times higher odds (confidence interval (CI): 2.583-9.480, P = <0.001) of being vaccinated as compared to Saudi patients. The odds of being vaccinated (CI: 1.284-2.882, P 0.002) were 1.924 times greater for males than females. Young patients aged 18-39 had 1.997 times higher odds (CI: 1.238-3.222, P 0.004) of being vaccinated than patients aged 60+ (Table 2).

**Table 2:** Binary logistic regression of vaccination status with the sociodemographic characteristics * Statistically significant at p < 0.05

Variables	B	S.E.	Wald	df	Sig.	Exp(B)	95% C.I. for EXP(B)	
							Lower	Upper
Nationality (Non-Saudi )	1.235	.305	16.901	1	.001*	4.583	2.583	9.480
Gender (Male)	.725	.161	20.306	1	.002*	1.924	1.284	2.882
Age group (18-39)	.692	.244	8.043	1	.004*	1.997	1.238	3.222
Age group (40-59)	.485	.263	3.389	1	.066	1.624	.969	2.721
Constant	-2.015	.248	65.757	1	.000	.133		

Table 3 reveals the clinical characteristics of the investigated patients. More than half of the patients (52.6%) presented with symptoms that were the indication for performing the COVID-19 nasopharyngeal swab; 25.8% were contacts of positive cases, 17.2% were both having symptoms and contact, whereas only a few (4.3%) had taken the swab as a routine prior to hospital measures such as surgery or diagnostic procedures. Almost two-thirds (63.3%) of cases had no underline medical conditions. However, 36.7% had at least one disease defined by the Centers for Disease Control and Prevention (CDC) as a risk factor for severe COVID-19 symptoms or complications. These conditions included bronchial asthma (4.3%), diabetes (3.7%), hypertension (2.9%), cancer (2.4%), as well as pregnancy (1.5%). In addition, 16.2% had multiple comorbidities, which were mainly diabetes with hypertension. Table 3 shows the significant differences, p-value <0.005; thus, the vaccinated group was mainly patients' contacts (28.9%) and those with no medical conditions (28.1%).

**Table 3:** Clinical characteristics Data are presented as numbers (n) and percentages (%). * Statistically significant at p < 0.05 DM: diabetes mellitus; HTN: hypertension

Variables		Total n = 1058	Not vaccinated n = 793 (75%)	Vaccinated n = 265 (25%)	Chi-square	p-value
		n (%)	n (%)	n (%)		
Indication for taking a swab	Symptoms	557 (52.6%)	419 (75.2%)	138 (24.8%)	8.848	0.031*
	Contact	273 (25.8%)	194 (71.1%)	79 (28.9%)		
	Symptoms & Contact	182 (17.2%)	138 (75.8%)	44 (24.2%)		
	Hospital Procedures	46 (4.3%)	42 (91.3%)	4 (8.7%)		
Medical Conditions	No	670 (63.3%)	482 (71.9%)	188 (28.1%)	8.831	0.003*
	Yes	388 (36.7%)	311 (80.2%)	77 (19.8%)		
	DM	39 (3.7%)	23 (59.0%)	16 (41.0%)		
	HTN	31 (2.9%)	17 (54.8%)	14 (45.2%)		
	Bronchial Asthma	45 (4.3%)	37 (82.2%)	8 (17.8%)		
	Oncology	25 (2.4%)	22 (88.0%)	3 (12.0%)		
	Multiple Comorbidities	171 (16.2%)	142 (83.0%)	29 (17.0%)		
	Pregnancy	16 (1.5%)	15 (93.8%)	1 (6.3%)		
	Other Diseases	61 (5.8%)	55 (90.2%)	6 (9.8%)		

Table 4 shows the significant difference in hospital admissions of COVID-19 patients between vaccinated and unvaccinated (p-value <0.001). Although the incidence rate of hospital admission had a lower percentage across both groups (12.9%), however, it was significantly lower among the vaccinated group (2.3%) compared to the unvaccinated (16.5%).

**Table 4:** Incidence of hospital admission of COVID-19 patients Data are presented as numbers (n) and percentages (%). * Statistically significant at p < 0.05

	Home Isolation	Hospital Admission	p-value
	n (%)	n (%)	
Not Vaccinated (n =793)	662 (83.5%)	131 (16.5%)	<0.001*
Vaccinated (n = 265)	259 (97.7%)	6 (2.3%)	
Total	921 (87.1%)	137 (12.9%)	

Table 5 shows the severity of the symptoms. Almost half of all investigated patients (48.3%) reported moderate symptoms, such as mild fever and cough, which required home isolation and regular phone calls for follow-up. A smaller proportion (1.3%) reported severe symptoms that required ICU admission such as shortness of breath. However, many cases (16.9%) had no symptoms (asymptomatic). The incidence of dead patients with COVID-19 was 1.9%. Moreover, Table 5 shows that both vaccinated and unvaccinated groups reported symptoms ranging from mild to death. However, there were significant differences in symptom severity among the vaccinated and unvaccinated groups, p-value <0.001. Among those who were vaccinated, only one patient (0.4%) died, one patient (0.4%) was admitted to the ICU, and one patient (0.4%) was admitted to the hospital isolation ward. On the other hand, among the unvaccinated group, 19 patients (2.4%) died, 13 patients (1.6%) were admitted to the ICU, and 99 patients (12.5.%) were admitted to the hospital isolation ward. The mild symptoms, such as sore throat and headache, were higher among vaccinated patients (35.5%) compared to the unvaccinated (17.7%). Furthermore, the percentage of free symptoms (asymptomatic) among vaccinated and unvaccinated was 23.0% and 14.9%, respectively. These results indicate that vaccination reduced the possibility of death by 2% and the severity of symptoms (hospital admission) by 13.3%, as well as increasing the probability of having mild symptoms by 17.8% and being free of symptoms by 8.1%

**Table 5:** Severity of COVID-19 symptoms Data are presented as numbers (n) and percentages (%). * Statistically significant at p < 0.05

	Asymptomatic	Mild (Home Isolation)	Moderate (Home Isolation)	Severe (Hospital Admission)	V. Severe (ICU Admission)	Death	Chi-square	p-value
	n (%)	n (%)	n (%)	n (%)	n (%)	n (%)		
Not Vaccinated n =793	118 (14.9%)	140 (17.7%)	404 (50.9%)	99 (12.5%)	13 (1.6%)	19 (2.4%)	78.353	<0.001*
Vaccinated n= 265	61 (23.0%)	94 (35.5%)	107 (40.4%)	1 (0.4%)	1 (0.4%)	1 (0.4%)		
Total	179 (16.9%)	234 (22.1%)	511 (48.3%)	100 (9.5%)	14 (1.3%)	20 (1.9%)		

In Table 6, a multivariable analysis using binary logistic regression was conducted to predict the relation between hospitalization and vaccination with adjustment for age, gender, and morbidities. It showed that unvaccinated COVID-19 patients are more likely to have a severe infection that leads to hospital admission, the ICU, or death than those vaccinated with a single dose of the vaccine.

**Table 6:** Binary logistic regression of hospitalization (severe disease) with the vaccination with adjustment of other variables *adjusted odds ratio; adjusted for gender, age, and morbidities

Variables	B	S.E.	Wald	df	Sig.	Exp (B)	95% C.I. for EXP (B)	
							Lower	Upper
Vaccination (Vaccinated)	-3.406	.731	21.715	1	.000	.033*	.008	.139
Gender (Male)	-.022	.219	.010	1	.920	.978	.637	1.503
Age			75.590	2	.000			
Age group (40-59)	.406	.394	1.060	1	.303	1.500	.693	3.249
Age group (60+)	2.140	.406	27.786	1	.000	8.499	3.835	18.832
Morbidities	1.002	.255	15.462	1	.000	2.723	1.653	4.487
Constant	-2.653	.370	51.544	1	.000	.070		

## Discussion

Since the beginning of vaccine production, people have raised concerns and worries over adverse events and risks associated with the COVID-19 vaccine. Factors such as knowledge about vaccines, possible risks, personal experiences, religious or cultural beliefs, and political motives, as well as social and economic status, determine the level of public trust in vaccines [12] 

Even if people do not develop COVID-19 symptoms after vaccination, they may still be infected with the virus and spread it to others. Understanding how effective vaccines are at preventing infection is, therefore, critical in predicting the likely impact of the vaccination program on the larger population. Multiple studies have been published on the effectiveness of vaccines against infection in healthcare workers, nursing home residents, and the general population. Vaccine effectiveness against symptomatic or asymptomatic SARS-CoV-2 infection was 74.7 percent among elderly nursing home residents who received two doses of a messenger RNA (mRNA) vaccine three weeks apart in the United States from March to May 2021 but dropped to 53.1 percent between June and July 2021, when the Delta variant was circulating [10]. For this study, all the vaccinated cases received only one dose of either Pfizer-BioNtech (BNT162b2) or AstraZeneca-Oxford (AZD1222) because the Saudi Ministry of Health postponed the second dose till it achieved complete coverage of the population. However, a similar study in Saudi showed a 92% protection rate of a single dose from the two vaccines [13]. For multiple doses, existing evidence suggests that longer intervals between doses increase vaccine effectiveness, and if this also applies to third doses, the administration interval must be considered. Simultaneously, third doses may be more reactogenic than previous doses, mainly if the recipient receives different vaccines for the initial and booster doses. Half-dose boosters or boosting with variant-targeted vaccines, both being researched, are appealing alternatives [14].

This study revealed a significant difference in symptoms' severity and, therefore, a difference in admission rates between the vaccinated and unvaccinated groups, p-value. Out of 265 of those who were vaccinated, only one patient (0.4%) expired, one patient (0.4%) was admitted to ICU, and one patient (0.4%) was admitted to the hospital isolation ward. On the contrary, among the 793 unvaccinated cases, 19 patients (2.4%) died, 17 patients (2.1%) were admitted to ICU, and 114 patients (14.4%) were admitted to the hospital isolation ward. It also showed that vaccinated patients mainly presented with mild symptoms such as cough, sore throat, headache, and mild fever; as a result, they were managed at home (35.5%), compared to the unvaccinated (17.7%). Furthermore, the percentage of asymptomatic cases among vaccinated and unvaccinated was 23.0% and 14.9%, respectively. Antonelli, Michela, et al. [15] reported similar results as symptoms of COVID-19 infection were less common in vaccinated than in unvaccinated participants, and the asymptomatic were more vaccinated than in the unvaccinated group. This increased incidence of asymptomatic or minimally symptomatic infection in vaccinated participants underlines the importance of individuals who interact with unvaccinated or clinically vulnerable groups (e.g., healthcare workers and social care workers) continuing to regularly take tests for SARS-CoV-2, even if vaccinated, in line with current UK testing guidelines [15]. The study also found that COVID-19 was less severe (regarding the number of symptoms in the first week of infection and the need for hospitalization) in participants after their first or second vaccine doses than in unvaccinated participants. 

This study covered the period from January 2021 to May 2021, before the emergence of the SARS-CoV-2 B.1.617.2 (Delta) variant [16], which affects vaccine efficacy. Multiple countries with high vaccination rates reported significantly reduced protection against the delta variant infection. In the United Kingdom, a study recently found that vaccine effectiveness was slightly lower against symptomatic disease with the B.1.617.2 (Delta) variant than with the B.1.1.7 (alpha) variant among adults that received two doses of the BNT162b2 vaccine (Comirnaty, Pfizer-BioNTech) (88.0% vs. 93.7%) or the ChAdOx1-S vaccine (also known as ChAdOx1 nCoV-19; Vaxzevria, AstraZeneca) (67.0% vs. 74.5%), administered over an extended interval of 12 weeks [17]. 

According to our study results, only 265 (25%) patients received the vaccine dose ≥14 days before illness onset or swab testing. The demographic characteristics of the investigated patients showed that their median age was 34 years (IQR 25-51 years), 48.6% were in the younger age group (18-39 years), and 59.5% were males. Multiple studies reported that men are more likely than women to be vaccinated, as men have lower trust in rumors and negative information about the COVID-19 vaccine, so they have a lower hesitancy to the vaccine [18,19].

Our study showed that the hospital admission rate had a lower percentage across both groups (12.9%). However, it was significantly lower among the vaccinated group (2.3%) compared to the unvaccinated (16.5%). A previous study showed low immune responses and vaccine effectiveness among persons in clinical risk groups, most notably those with immunosuppression [20,21]. The United Kingdom and other countries already recommend a third dose of the vaccine against SARS-CoV-2 for all adults as part of their primary immunization course [7,22]. Johnson, Amelia G et al. [16] reported consistent results as unvaccinated persons had 13.9 and 53.2 times the risks for SARS-CoV-2 infection and death, respectively, in comparison with fully vaccinated persons who had booster doses, and 4.0 and 12.7 times the risks compared with fully vaccinated persons without booster doses. When the Omicron variant emerged in December 2021, the incidence rate ratio decreased to 4.9 for fully vaccinated persons with booster doses and 2.8 for those without booster doses, relative to October-November 2021. 

Considering the transmissible nature of COVID-19, the epidemiological data report fluctuation, especially in the mortality rate. In Saudi Arabia, the incidence of COVID-19 and the mortality rate have been gradually declining, reflecting successful preventive measures and effective healthcare practices and treatment protocols. In this study, the COVID-19 vaccine reduced the possibility of death by 2% and the severity of symptoms (hospital admission) by 15.3%. A study by Bernal et al. showed evidence of the effectiveness of the Pfizer-BioNTech BNT162b2 and Oxford-AstraZeneca ChAdOx1-S vaccinations against symptomatic COVID-19 disease, hospitalization, and death in the older population [8]. The vaccinated group had a 44% decreased risk of hospitalization and a 51% lower risk of death than the unvaccinated group. High levels of protection (over 90%) are also seen against mortality with three types of vaccines and against both the Alpha and Delta variants in many other studies [23-25]. 

The limitation of this study is that it covered a period before the spread of the Delta variant among the study population. Further studies are recommended for any new strain of the virus. The generalizability of the results is limited, so wide national studies are needed, including larger samples and assessing the multiple doses effects of the vaccines.

## Conclusions

This retrospective cohort demonstrates a significant difference in symptom severity, hospitalization, and death between the vaccinated and the unvaccinated with COVID-19 vaccines before the spread of the Delta variant (B.1.617). It showed that unvaccinated COVID-19 patients are more likely to have a severe infection that leads to hospital admission, the ICU, or death. A single dose of either Pfizer-BioNTech or Oxford-AstraZeneca lowers the possibility of death by 2% and hospital admission by 13.3%, as well as increases the probability of mild or asymptomatic infection with SARS-CoV-2 by 17.8% and 8.1%, respectively.

For more generalizable results, nationwide studies are needed to assess the effect of multiple doses of the vaccines on different variants of SARS-CoV-2.
